# Characterization of Two-Pore Channel 2 by Nuclear Membrane Electrophysiology

**DOI:** 10.1038/srep20282

**Published:** 2016-02-03

**Authors:** Claire Shuk-Kwan Lee, Benjamin Chun-Kit Tong, Cecily Wing-Hei Cheng, Harry Chun-Hin Hung, King-Ho Cheung

**Affiliations:** 1 School of Biomedical Sciences, LKS Faculty of Medicine, University of Hong Kong, Hong Kong, China; 2 Research Centre of Heart, Brain, Hormone and Healthy Aging, LKS Faculty of Medicine, University of Hong Kong, Hong Kong, China

## Abstract

Lysosomal calcium (Ca^2+^) release mediated by NAADP triggers signalling cascades that regulate many cellular processes. The identification of two-pore channel 2 (TPC2) as the NAADP receptor advances our understanding of lysosomal Ca^2+^ signalling, yet the lysosome is not amenable to traditional patch-clamp electrophysiology. Previous attempts to record TPC2 single-channel activity put TPC2 outside its native environment, which not reflect TPC2’s true physiological properties. To test the feasibility of using nuclear membrane electrophysiology for TPC2 channel characterization, we constructed a stable human TPC2-expressing DT40TKO cell line that lacks endogenous InsP_3_R and RyR (DT40TKO-hTPC2). Immunostaining revealed hTPC2 expression on the ER and nuclear envelope. Intracellular dialysis of NAADP into Fura-2-loaded DT40TKO-hTPC2 cells elicited cytosolic Ca^2+^ transients, suggesting that hTPC2 was functionally active. Using nuclear membrane electrophysiology, we detected a ~220 pS single-channel current activated by NAADP with K^+^ as the permeant ion. The detected single-channel recordings displayed a linear current-voltage relationship, were sensitive to Ned-19 inhibition, were biphasically regulated by NAADP concentration, and regulated by PKA phosphorylation. In summary, we developed a cell model for the characterization of the TPC2 channel and the nuclear membrane patch-clamp technique provided an alternative approach to rigorously investigate the electrophysiological properties of TPC2 with minimal manipulation.

Modulation of the cytoplasmic Ca^2+^ concentration elicits universal signals that regulate processes involved in life and death^1^. Three Ca^2+^ -mobilizing messengers have been identified: inositol trisphosphate (InsP_3_), cyclic ADP-ribose (cADPR), and nicotinic acid adenine dinucleotide phosphate (NAADP). It is well established that InsP_3_ binds the InsP_3_ receptor (InsP_3_R), leading to Ca^2+^ release from the endoplasmic reticulum (ER), whereas cADPR may mobilize ER Ca^2+^ by activating the ryanodine receptor (RyR)^2^,^3^. Unlike the other two messengers that target the ER, NAADP, the most potent Ca^2+^ mobilizing messenger, was discovered as a contaminant of β-NADP^+^ by Lee^4^ and has been subsequently demonstrated to mobilize Ca^2+^ from acidic lysosomal Ca^2+^ stores^4^,^5^. Although the intracellular location of these receptor channels was not amenable to conventional patch-clamp electrophysiology, the channel functions and ligand regulations of InsP_3_R and RyR have been extensively studied [reviewed in]^6^,^7^. Nevertheless, the molecular identity of the NAADP receptor was unresolved until the recent discovery that a novel lysosomal cation channel family (two-pore channels (TPCs) 1 and 2 in human) is the putative receptor responsible for NAADP activation^8^,^9^,^10^. The idea that TPC was the NAADP receptor was supported by ample evidence: (i) NAADP-mediated lysosomal Ca^2+^ release is dependent on TPC expression^8^,^9^,^11^; (ii) TPCs exhibited a biphasic NAADP concentration-response curve^8^,^9^,^10^ and were sensitive to Ned-19, a selective antagonist selective of NAADP-activated Ca^2+^ mobilization^8^,^10^; and, most importantly, (iii) electrophysiology demonstrated that TPC is the Ca^2+^ -permeable channel activated by NAADP^10^,^12^.

Single-channel currents of human TPC2 (hTPC2) have been successfully recorded by reconstituting purified protein into an artificial lipid bilayer^10^,^13^ or by retargeting hTPC2 protein to the plasma membrane by mutating the dileucine lysosomal signalling motif^12^. Although these electrophysiological studies provided examples of the biophysical properties of the hTPC2 channel mediated by NAADP, these approaches have their drawbacks. Furthermore, recent reports from vacuolin-1 enlarged whole-lysosome recording suggested that the opening of TPC2 channel is not mediated by NAADP but phosphatidylinositol 3,5-bisphosphate [PI(3,5)P_2_]^14^,^15^; this finding prompts the need for the development of a novel methodology for characterizing the hTPC2 channel in its native membrane. In this regard, we generated a stable DT40 cell line expressing hTPC2 (DT40TKO-hTPC2) that lacked both functional InsP_3_R and RyR (DT40TKO)^16^,^17^ to eliminate the influences by these intracellular Ca^2+^ release channels. Using the nuclear membrane patch-clamp technique, we detected a ~220 pS single-channel current activated by NAADP with K^+^ as the permeant ion. The detected single-channel recordings displayed a linear current-voltage relationship, were inhibited by Ned-19, were biphasically regulated by NAADP concentrations, and its channel open probability (*P*_o_) was regulated by PKA phosphorylation.

Taken together, we developed a cell model with minimal manipulation that, combined with nuclear membrane electrophysiology^18^, enabled us to rigorously investigate the biophysical properties of the TPC2 channel in its native membrane.

## Results

### Generation of a stable hTPC2-expressing DT40TKO line

**Figure 1:**
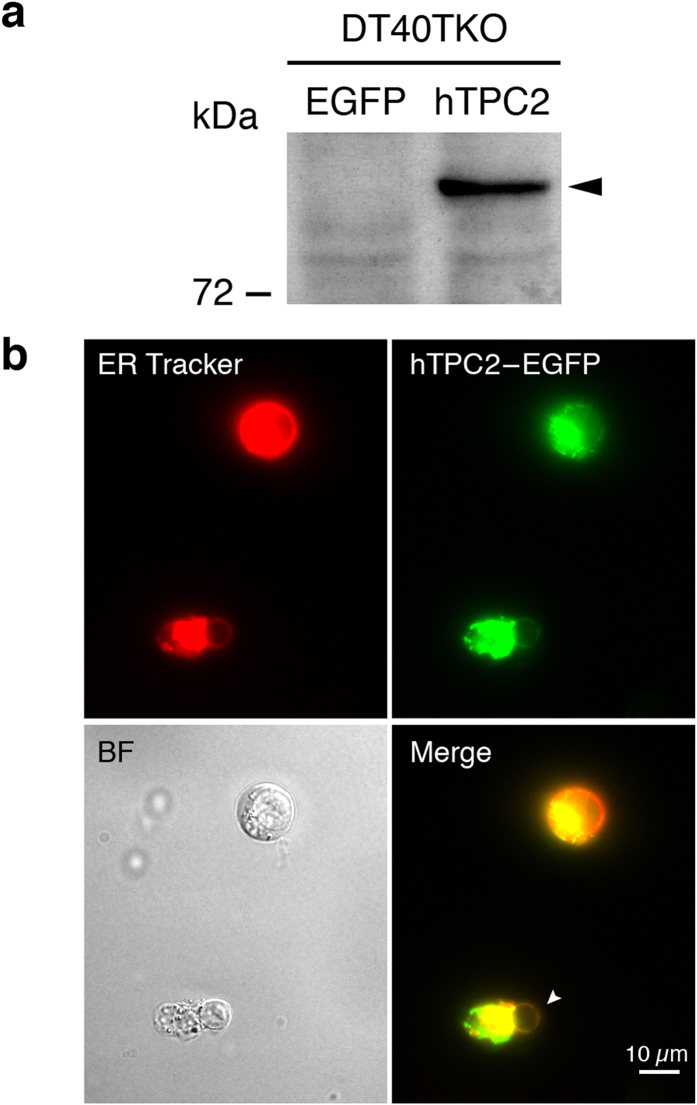
Construction of the stable human TPC2 (hTPC2)-expressing cell line, DT40TKO-hTPC2. (**a**) Expression of hTPC2 in DT40TKO-EGFP (lane 1) and DT40TKO-hTPC2 (lane 2) cells. Twenty-five μg of cell lysate were loaded in each lane; arrowhead indicates the immunoreactive band of TPC2 protein. (**b**) Fluorescent microscopy of GFP-tagged hTPC2 protein in intact and ruptured DT40TKO-hTPC2-GFP cells counterstained with ER tracker. Arrow indicates the expression of GFP-tagged hTPC2 protein on the nuclear envelope of a ruptured DT40TKO cell.

**Figure 2:**
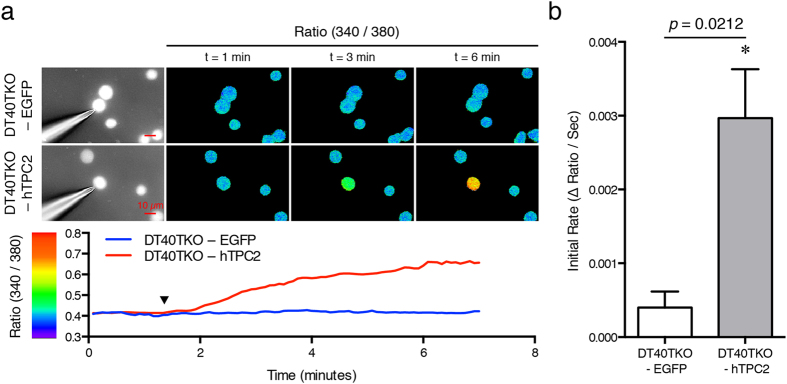
Calcium imaging of hTPC2 expressed in DT40TKO cells. (**a**) Fura-2 ratiometric images showed changes in the cytosolic Ca^2+^ concentrations of cells dialysed with 10 nM NAADP in the whole-cell configuration at different time points (upper panel). Representative Fura-2 fluorescent ratio in response to pipette-dialysed NAADP in DT40TKO-EGFP or DT40TKO-hTPC2 cells. Arrowhead indicates the achievement of whole-cell configuration (lower panel). (**b**) Summary of the initial rate of the Fura-2 fluorescent ratio in cells dialysed with 10 nM NAADP. Bars are presented as mean ± SEM from 3 experiments and data are compared by Student’s *t*-test.

The single-channel properties of hTPC2 were successfully revealed by the use of lipid bilayer electrophysiology and retargeting channel proteins to the plasma membrane^10^,^12^,^13^; these approaches, however, have major drawbacks that might not truly reflect the biophysical properties of hTPC2 in its native membrane environment. Human TPC2 is predominantly expressed in the late endosome, lysosome, and ER membrane^11^; therefore, we tested whether nuclear membrane electrophysiology^18^,^19^ could be employed to characterize the electrophysiological properties of the hTPC2 channel. As proposed by Cancela and others^12^,^20^, NAADP initiates local Ca^2+^ release from the TPC2 channel that is amplified subsequently by ER Ca^2+^ releasing channel through the Ca^2+^ -induced Ca^2+^ release. To prevent the influence of other intracellular Ca^2+^ release channels (InsP_3_Rs and RyRs), a subline of InsP_3_R deficient chicken B lymphocyte, DT40TKO was used^16^,^17^. This cell line is insensitive to anti-IgM ligation and caffeine stimulation (Supplementary Fig. 2). We established a stable hTPC2 expressing DT40TKO cell line by retroviral infection (DT40TKO-hTPC2). Western blot was used to confirm hTPC2 expression in the DT40TKO-hTPC2 cell line; as shown in Fig. 1a, a band of ~83 kDa was detected by the anti-hTPC2 antibody in cell lysates prepared from the DT40TKO-hTPC2 line but not from the control EGFP-expressing cells. To confirm the location of the expressed hTPC2, a GFP-tagged, hTPC2-expressing DT40TKO cell line (DT40TKO-hTPC2-GFP) was generated. Fluorescence microscopy and ER-Tracker Blue-White DPX counterstaining revealed that hTPC2 was expressed in the ER and the nuclear membrane (arrowhead) of an exposed nucleus (Fig. 1b). To verify whether the expressed hTPC2 in DT40TKO-hTPC2 cells formed functional channels, we dialysed NAADP to a DT40TKO-hTPC2 cell using a patch pipette in the whole-cell configuration and monitored the changes in cytosolic [Ca^2+^]. After the whole-cell configuration was achieved, hTPC2-expressing cells showed potentiated increases in cytosolic [Ca^2+^]; in contrast, this NAADP-mediated cytosolic [Ca^2+^] increases was absent in control EGFP-expressing cells (Fig. 2a). The rate of NAADP-elicited Ca^2+^ response in hTPC2-expressing cells was significantly higher than those of the control cells (*p* = 0.0212, n = 3 by unpaired Student’s *t*-test), as shown in Fig. 2b. Taken together, our data demonstrate that functional and stably-expressed hTPC2 localized to the ER and nuclear membrane was generated in DT40TKO cells that lacked functional InsP_3_R and RyR.

### NAADP-activated hTPC2 single-channel current in nuclear envelope

**Figure 3:**
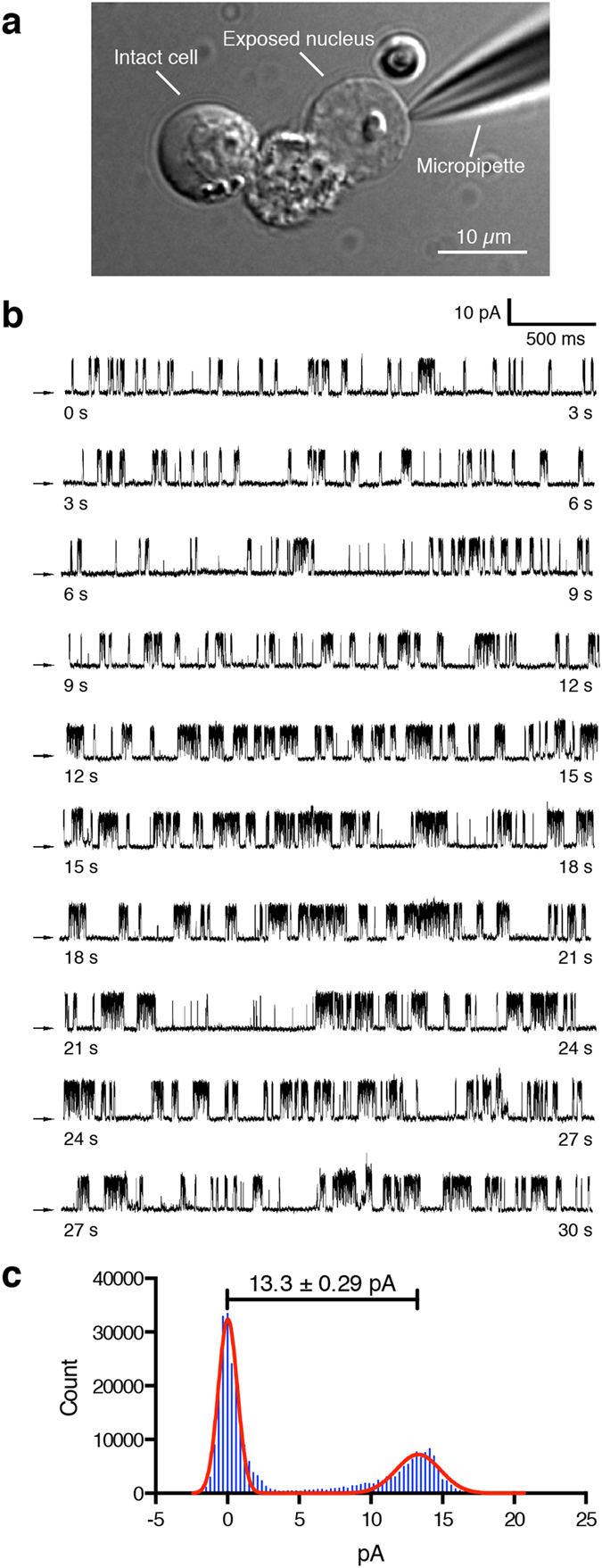
NAADP-activated single-channel activity in isolated nuclei of DT40TKO-hTPC2 cells. (**a**) Brightfield DIC micrograph showed both intact and ruptured DT40TKO-hTPC2 cells. The glass pipette was positioned on the surface of the nuclear envelope for electrophysiological measurement of the hTPC2 channel by nuclear membrane patch-clamp. (**b**) A 30-second representative current trace detected from an isolated DT40TKO-hTPC2 nucleus activated by 10 nM NAADP in symmetric 140 mM K^+^ solutions. Arrows indicate zero current level and the traces were recorded at +60 mV. (**c**) All-points histogram depicts the current amplitudes of the open and closed states of the NAADP-activated TPC2 single channel from isolated DT40TKO-hTPC2 nuclei recorded at +60 mV. The open-state amplitude was 13.3 ± 0.29 pA, which is equivalent to ~220 pS.

Ca^2+^ imaging techniques have revealed that NAADP mobilizes lysosomal Ca^2+^ via the activation of TPC2 channels^9^,^11^ and Pitt *et al.* demonstrated, in planar lipid bilayer, that NAADP dose-dependently activates the hTPC2 channel^10^. To test if NAADP can directly activate hTPC2 expressed on the nuclear envelope, we performed nuclear membrane electrophysiology on the DT40TKO-hTPC2 in the “on-nucleus” configuration. Nuclei can be obtained from DT40TKO-hTPC2 cells by mechanical rupture^18^,^21^ (Fig. 3a); gigaohm seals were readily achieved in most of the exposed nuclear membranes. In symmetrical 140 mM KCl (K^+^ as the charge carrier) with 10 nM NAADP, single-channel currents were detected in ~30% of the nuclear patches (n = 412 patches). A 30-second representative current trace of our nuclear patch in symmetric K^+^ recorded at +60 mV is shown in Fig. 3b. In the presence of 10 nM NAADP in the pipette solution, hTPC2 single-channel currents with a channel open probability (*P*_o_) of 0.39 ± 0.07 (n = 3) were detected from nuclei isolated from DT40TKO-hTPC2 cells; this NAADP-activated single-channel current was not observed when we patched the DT40TKO-EGFP nuclei (data not shown). An amplitude histogram revealed that the detected hTPC2 channels had current amplitudes similar to those obtained from the planar lipid bilayer study (Fig. 3c; current amplitude at +60 mV was 13.3 ± 0.29 pA, resulting in a conductance of ~220 pS). Our results show that NAADP activated the hTPC2 channels expressed in the nuclear membrane of DT40TKO-hTPC2 cells and that nuclear membrane electrophysiology is suitable for single-channel recording of NAADP-activated hTPC2 channels.

### TPC2 channel on the nuclear membrane is permeable to K^+^ and Cs^+^

**Figure 4:**
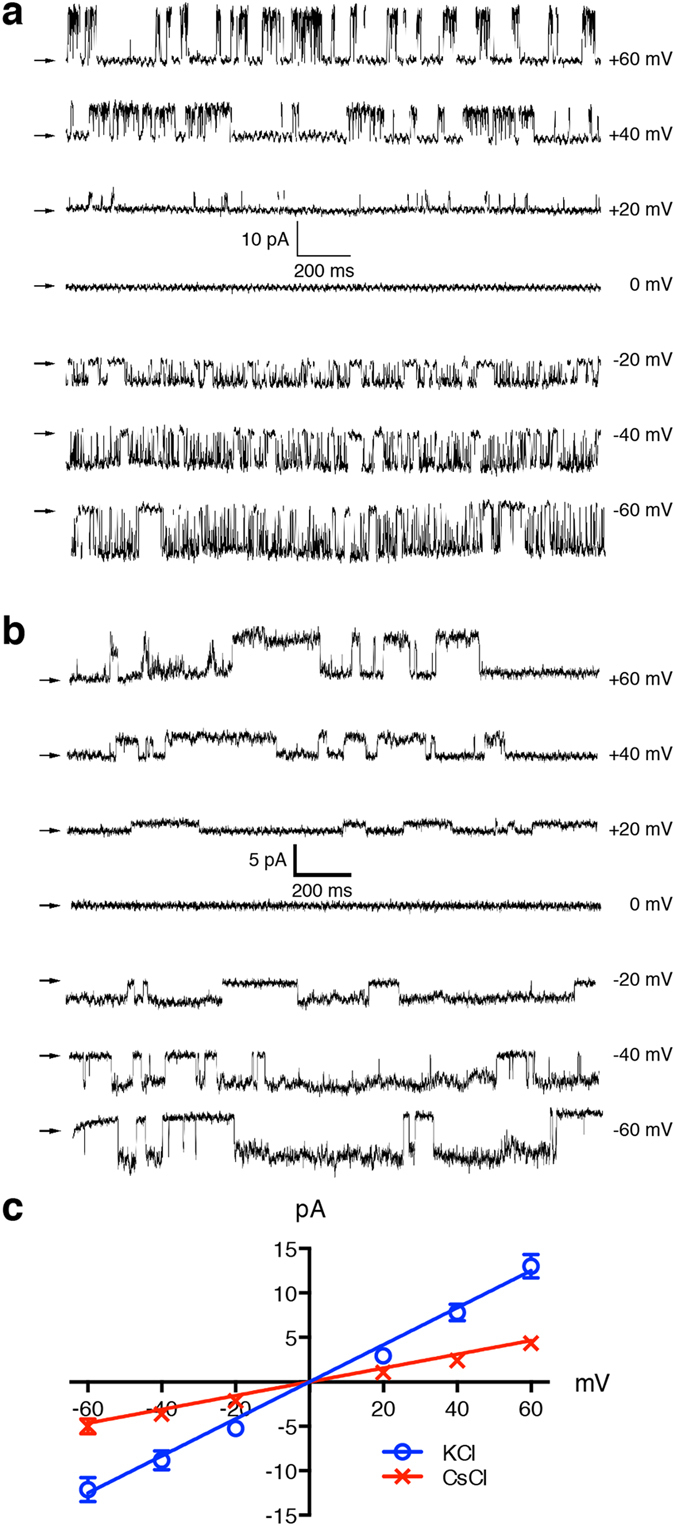
Current-voltage relationship and ion selectivity of hTPC2 channels expressed on DT40TKO-hTPC2 nuclei. Representative current traces recorded in symmetrical K^+^ (**a**) and Cs^+^ (**b**) at different voltages, as tabulated. Traces were obtained at holding potentials from −60 mV to +60 mV with 20−mV increments. (**c**) Mean ± SEM current-voltage relationship were constructed for both KCl and CsCl experiments (n = 5 patches).

As demonstrated in the reconstituted planar lipid bilayer, hTPC2 forms a large conductance, cation-selective channel permeable to monovalent and divalent ions, similar to other intracellular Ca^2+^ release channels^10^,^12^. We performed analogous experiments by nuclear membrane patch to investigate whether the conductance and permeation properties observed by nuclear membrane patch resemble those observed in the planar lipid bilayer experiments. Current/voltage (*I*/*V*) relationships were determined using 2 μM Ca^2+^ in the pipette solution, with symmetrical K^+^ or Cs^+^ as the permeant ion (140 mM pipette:bath). Fig. 4a (K^+^) and Fig. 4b (Cs^+^) depict representative current sweeps that lasted for 2 s each, with membrane potential steps from −60 mV to +60 mV in 20−mV increments. The *I*/*V* relationships generated from these experiments are illustrated in Fig. 4c. The detected hTPC2 channels, in both symmetric K^+^ and Cs^+^, exhibited an ohmic *I*/*V* relationship with no discernible voltage dependence. The slope conductance of K^+^ as the charge carrier was 208.4 ± 20.7 pS, which resembles those reported in the lipid bilayer^10^,^12^; in comparison, the slope conductance of Cs^+^ was 77.5 ± 13.6 pS (Fig. 4c). The conductance and permeation properties of the hTPC2 channel recorded from the nuclear membrane are similar to those reported in previous studies with the lipid bilayer and hTPC2 retargeting to the plasma membrane^10^,^12^.

### NAADP biphasically regulates hTPC2 channel activity

**Figure 5:**
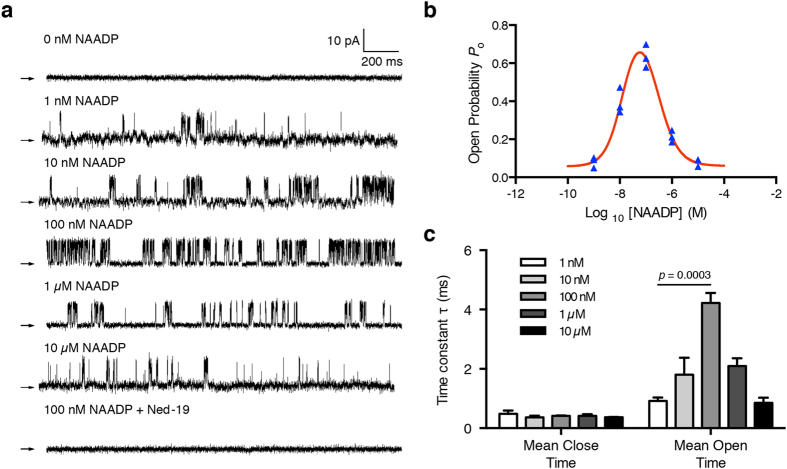
NAADP elicited biphasic regulation of the hTPC2 channel expressed on DT40TKO-hTPC2 nuclei. (**a**) Representative hTPC2 single-channel current traces activated by various NAADP concentrations, as tabulated, at +60 mV. No current was detected when NAADP was excluded from the pipette solution (n = 20 patches). The NAADP-activated single-channel current was inhibited when 1 μM Ned-19 was included in the pipette solution (n = 20 patches). Arrows indicate zero current level. (**b**) Relationship between hTPC2 *P*_o_ and [NAADP]. All data were recorded at +60 mV and data were summarized as mean ± SEM from 3 individual recordings at each [NAADP]. (**c**) Summary of effects of [NAADP] on hTPC2 channel mean open and mean closed times. Data were compared to 1 nM NAADP by one-way ANOVA with Dunnett’s post hoc test. Individual dwell time analysis at different [NAADP] was presented in Supplementary Fig. 3.

NAADP dose-dependently regulates lysosomal Ca^2+^ release and two distinct NAADP binding sites have been proposed^20^,^22^. To investigate this unique NAADP dependence characteristic, we performed nuclear membrane patches on DT40TKO-hTPC2 nuclei and included different concentrations of NAADP in the pipette solution. As shown in the representative traces in Fig. 5a, no TPC2 channel activity was detected when NAADP was omitted from the pipette solution or when 1 μM Ned-19, a NAADP antagonist, was included in the pipette solution with 100 nM NAADP. With the presence of NAADP in the pipette solution, hTPC2 single channel *P*_o_ increased dose-dependently from 0.08 ± 0.03 (1 nM NAADP) to 0.39 ± 0.07 (10 nM NAADP) and reached a maximum *P*_o_ of 0.63 ± 0.06 (100 nM NAADP; Fig. 5a,b; summarized from 3 different single channel patches at each NAADP concentration). Human TPC2 single-channel *P*_o_ started to decrease when the NAADP concentration was further increased (Fig. 5a,b). Human TPC2 *P*_o_ was 0.21 ± 0.03 when 1 μM NAADP was included in the pipette solution, whereas *P*_o_ decreased further to 0.07 ± 0.03 when 10 μM NAADP was added to the pipette solution (Fig. 5a,b). To gain mechanistic insights into the effects of NAADP on hTPC2 channel *P*_o_, we performed dwell-time analyses on the recorded hTPC2 currents. In sub-micromolar concentrations, NAADP increased hTPC2 *P*_o_ by increasing the channel open time, whereas the channel closed time remain unaltered (Fig. 5c & Supplementary Fig. 3a,b,c); micromolar concentrations of NAADP inhibited hTPC2 channel activity by decreasing the channel open time (Fig. 5c & Supplementary Fig. 3d,e). Our results show that the unique bell-shaped NAADP dependence of hTPC2 channel activity is preserved when the channel is expressed on the nuclear membrane. NAADP affects hTPC2 channel *P*_o_ by regulating the stability of the channel’s open state.

### hTPC2 channel activity is regulated by protein kinase A phosphorylation

**Figure 6:**
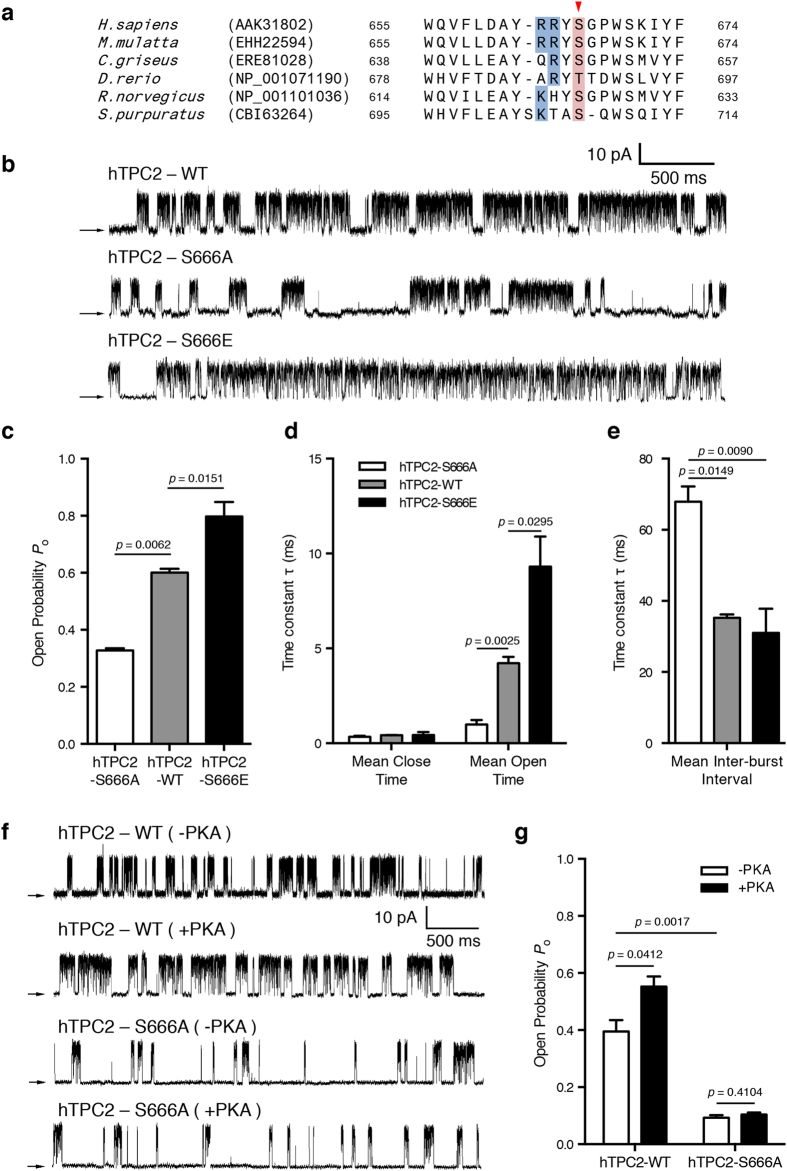
PKA phosphorylation increased hTPC2 channel *P*_o_. (**a**) Sequence alignments of mammalian TPC2 C-terminus highlighted a conserved putative PKA phosphorylation site with a consensus sequence of RRXS/T. (**b**) Representative current traces recorded from isolated nuclei of wild-type hTPC2, S666A, and S666E DT40TKO cell lines activated by 100 nM NAADP. Arrows indicate zero current level and the data were recorded at +60 mV. (**c**) Summary of NAADP-elicited TPC2 *P*_o_ detected in wild-type hTPC2, S666A, and S666E cell lines. (**d**) Summary of effects of PKA phosphorylation on hTPC2 channel mean open and closed times. Data were summarized as mean ± SEM from 3 individual experiments and were compared to wild-type hTPC2 (Dunnett’s post hoc test following a one-way ANOVA in (**c,d**). (**e**) Summary of effects of PKA phosphorylation on mean inter-burst interval of hTPC2 channel. Data were summarized as mean ± SEM from 3 individual experiments and were analysed by one-way ANOVA with Tukey’s post hoc test. Individual dwell time analysis was presented in Supplementary Fig. 5. (**f**) Representative current traces from isolated nuclei of wild-type hTPC2 and S666A stimulated by 10 nM NAADP together with or without PKA catalytic subunit (50 nM) in the pipette solution. (**g**) Summary of effects of PKA catalytic subunit on single-channel *P*_o_ detected from wild-type hTPC2 and hTPC2-S666A nuclei. Data were summarized as mean ± SEM from 3 individual experiments and were analysed by one-way ANOVA with Tukey’s post hoc test.

Apart from regulation by physiological ligands and Ca^2+^, the channel activity of InsP_3_Rs and RyRs are modulated by different protein kinases^6^,^7^. We analysed the TPC2 protein sequences and found a putative protein kinase A (PKA) phosphorylation site at position 666 in human, which is conserved in several other mammals (Fig. 6a). We therefore investigated the influence of PKA phosphorylation on hTPC2 channel activity using DT40TKO stable cell lines expressing either phosphomimetic (S666E) or unphosphorylatable (S666A) mutants. Western blot analysis confirmed the expression of TPC2 proteins from cell lysates isolated from wild-type or mutant-expressing cells (Supplementary Fig. 4) and suggests that PKA phosphorylation of hTPC2 did not alter hTPC2 expression. As shown in the representative single-channel current traces in Fig. 6b, channel *P*_o_ was significantly increased in the phosphomimetic S666E mutant cell line (*P*_o_ = 0.80 ± 0.09; n = 3) as compared to wild-type hTPC2-expressing cells (*P*_o_ = 0.63 ± 0.06; n = 3) (*p* = 0.0151), suggesting that PKA phosphorylation of hTPC2 augments its activity; in contrast, the unphosphorylatable S666A mutation decreased channel activity (*P*_o_ = 0.33 ± 0.01 vs 0.63 ± 0.06 in wild-type, *p* = 0.0062). Average changes in channel *P*_o_ and channel open and closed times in wild-type and mutant-expressing cells are shown in Fig. 6c,d and Supplementary Fig. 5. To characterize how PKA phosphorylation affects hTPC2 channel gating, burst analysis was performed using a *T*_c_ (the time which separates inter-burst closures from intra-burst closures) of 4 ms; this value was determined from analysis of wild-type TPC2 closed-time histograms containing a single channel studied in the presence of 100 nM NAADP (Supplementary Fig. 1). Figure 6e shows the effect of PKA phosphorylation on channel inter-burst duration. Cells with the S666A mutation showed significantly increased inter-burst duration relative to both wild-type (Fig. 6e; 67.89 ± 4.30 *vs* 35.23 ± 0.98, *p* = 0.0149) and S666E (Fig. 6e; 67.89 ± 4.30 *vs* 31.03 ± 6.79, *p* = 0.009). To further validate PKA phosphorylation enhances TPC2 channel activity, we patched DT40TKO-hTPC2 nuclei with the addition of PKA catalytic subunit in the pipette solution. With the presence of PKA catalytic subunit and 10 nM NAADP in the pipette, hTPC2 channel *P*_o_ was increased significantly as compared to those in the absence of PKA (Fig. 6f, *P*_o_: 0.55 ± 0.04 *vs* 0.39 ± 0.04, *p* = 0.0412). Conversely, channel *P*_o_ in DT40TKO-hTPC2-S666A did not affected by PKA phosphorylation (*P*_o_: 0.11 ± 0.01 *vs* 0.09 ± 0.01, *p* = 0.410) but lower than those in wild-type DT40TKO-hTPC2 (*P*_o_: 0.09 ± 0.01 *vs* 0.39 ± 0.04, *p* = 0.002; Fig. 6f,g). Our data suggest PKA phosphorylation of hTPC2 results in significantly increased *P*_o_ with altered channel open durations, whereas dephosphorylation decreases *P*_o_ by shortening the channel open time (Fig. 6d) and increasing the inter-burst duration (Fig. 6e).

## Discussion

Ever since the TPC2 channel was proposed to be the NAADP-gated lysosomal Ca^2+^ release channel, characterization of TPC2′s electrophysiological properties has been one of the field’s top priorities. Macroscopic electrophysiological properties of TPC2 have been investigated by patch clamping of vacuolin enlarged lysosome^14^,^15^,^23^, whereas microscopic TPC2 single-channel currents were recorded by reconstituting immunopurified TPC2 protein into artificial bilayer^10^,^13^, or by patch clamping of TPC2 channel re-targeted to the plasma membrane^12^. TPC2 properties detected by these approaches may not reflect their real physiological conditions. The whole lysosome patch clamp approach used vacuolin to induce fusion of endosomes and lysosomes^24^. Whether this chemical-induced endosome/lysosome fusion affects TPC2 functions and the luminal ion compositions are uncertain. These may explain why data from this approach are controversial to those obtained from bilayer experiments [activated by NAADP *vs* PI(3.5)P_2_ and non-selective *vs* Na^+^ -selective^25^,^26^]. Furthermore, evidence has been suggested that NAADP may bind to accessory proteins to activate the TPCs^27^,^28^. The bilayer method requires tedious solubilization and purification procedures; therefore, the accessory protein required for TPC activation may not be able to reconstitute into the bilayer, which may explain why the maximum TPC2 *P*_o_ recorded from the bilayer method was relatively low^10^. While the plasma retargeting method of hTPC2 successfully demonstrated hTPC2 single-channel activity in the plasma membrane, the maximum detected *P*_o_ was also low in this system. Moreover, the plasma retargeting approach involves genetic modification of the channel protein at its N-terminus^12^. In a separate study by the same research team, they demonstrated that the N-terminus of TPC1, and possibly TPC2, is critical for NAADP binding^29^,^30^. Genetic modification of the channel at the N-terminus may adversely affect the physiological properties of the TPC channel, particularly its regulation by NAADP. To overcome these limitations and uncouple the influence of other intracellular Ca^2+^ release channels, we generated a stable hTPC2-expressing cell line in a sub-line of DT40 (DT40TKO) that lacks functional InsP_3_R and RyR (Supplementary Fig. 2) and tested the feasibility of using nuclear membrane patch-clamp to characterize the TPC2 channel in its native membrane environment. As shown by immunocytochemistry and Ca^2+^ imaging, we demonstrated that hTPC2 is expressed in the nuclear envelope of DT40TKO cells and our cell model is functionally responsive to intracellular NAADP dialysis (Figs 1 & 2).

Using nuclear membrane electrophysiology in the “on-nucleus” configuration, we demonstrated hTPC2 single-channel recordings without any genetic modification. Inclusion of 10 nM NAADP elicited a single-channel current with ~220 pS conductance when symmetrical K^+^ was the permeant ion (Fig. 3). Similar to the bilayer result, we observed a delayed onset of NAADP-activated channel activity (as shown in the slow Ca^2+^ mobilization kinetics in Fig. 2 and progressive increases of *P*_o_ in Fig. 3). As proposed, NAADP may interact with an unidentified accessory protein associated with the TPC protein complex instead of directly binding to the hTPC channel^27^,^28^. Our data supports the suggestion that NAADP needs to interact with the accessory protein to fully activate the hTPC2 channel.

The hTPC2 currents detected by nuclear membrane patch-clamp had many similar biophysical properties to those detected by bilayer methods^10^,^13^. NAADP activates the opening of the hTPC2 channel, which is sensitive to Ned-19 inhibition (Fig. 5). In Fig. 4, the open channel showed a linear *I/V* relationship and permeable to both K^+^ and Cs^+^, with slope conductances of 208.4 ± 20.7 pS and 77.5 ± 13.6 pS, respectively, suggesting that the hTPC2 channel is more permeable to K^+^. Controversial studies suggested that the TPC2 channel is a Na^+^ channel activated by PI(3,5)P_2_ but not NAADP^14^,^15^. Although we did not study Na^+^ permeability using nuclear membrane electrophysiology (due to the low solubility of PI(3,5)P_2_ in pipette solution), our permeability results and others have clearly demonstrated that hTPC2 is a non-selective cation channel activated by NAADP. Nevertheless, the use of nuclear membrane patch-clamp for the investigation of hTPC2 has its limitations. All our hTPC2 current traces were recorded in the “on-nucleus” configuration; the luminal nuclear environment could not be readily controlled under such configuration. As the sensitivity and affinity of hTPC2 to NAADP has been demonstrated to be dependent on luminal [Ca^2+^] and pH, respectively^10^,^13^, further nuclear membrane patches in the “luminal-side-out” and “cytoplasmic-side-out” configurations^18^,^19^,^31^ are required to fully characterize the electrophysiological properties of hTPC channels.

Nonetheless, the “on-nucleus” nuclear membrane patch of hTPC2 can provide some mechanistic insights into the regulation of channel activity by NAADP. Like the data from Ca^2+^ imaging studies, our nuclear membrane patch recapitulated the unique bell-shaped regulation of hTPC2 channel activity by [NAADP]. Nanomolar NAADP concentrations led to channel activation, whereas micromolar NAADP concentrations inactivated the channel (Fig. 5a,b). From the channel dwell time analyses (Fig. 5c & Supplementary Fig. 3), the increase in hTPC2 *P*_o_ as [NAADP] increased was accomplished by a marked increase in the time constant of the open time. While further [NAADP] increases resulted in significantly decreased mean channel open time, NAADP had no significant effect on the channel closed time constant. These results indicate that NAADP modulates the stability of the channel’s open state, consistent with the proposal that NAADP binds its receptor at two distinct binding sites^20^,^22^, a high-affinity activation site and a low-affinity inactivation site.

The channel activities of InsP_3_R and RyR are critically regulated by phosphorylation^6^,^7^; however, phosphorylation of TPCs by kinases has never been investigated. We identified a putative PKA phosphorylation site in hTPC2 and a phosphomimetic mutation showed significant increase in channel *P*_o_ (Fig. 6c). The augmentation of channel *P*_o_ by PKA phosphorylation is caused by stabilization of the channel’s open state, possibly due to the shortening of inter-burst intervals (Fig. 6e). Although the inter-burst intervals in the S666E mutant were not significantly different than those of wild-type hTPC2-expressing cells, a one-fold difference in inter-burst interval was observed between the S666E and S666A mutants. Furthermore, wild-type hTPC2 channel *P*_o_ was significantly increased with the presence of PKA catalytic subunit while the S666A mutation remained unaffected (Fig. 6f,g). These data suggests that the wild-type hTPC2 channel is partially phosphorylated which is agreed with our *in vitro* PKA activation assay (Fig. 6f,g). Further investigations on NAADP-induced Ca^2+^ release by imaging and single-channel patch-clamp experiments in a broad range of NAADP concentration may provide detail mechanistic insights into the regulation of hTPC2 by PKA phosphorylation.

In summary, the permeation and conducting properties of hTPC2 detected by nuclear membrane patch-clamp are consistent with those recorded in the lipid bilayer experiments. More importantly, our approach demonstrated the unique bell-shaped regulation of hTPC2 channel activity by [NAADP] and that channel activity is modulated by PKA phosphorylation at position S666. The application of the nuclear membrane patch-clamp technique to a DT40TKO-hTPC2 cell provides a robust system to characterize the electrophysiological properties of the hTPC2 channel in native nuclear membrane with minimal artificial manipulations.

## Methods

### Generation of stable human TPC2 (hTPC2)-expressing cell lines

The cDNA encoding hTPC2^9^ was kindly provided by Professor Antony Galione (University of Oxford, Oxford, England). The hTPC2 cDNA was sub-cloned into pΔMX-IRES-EGFP^32^ at the EcoRI and NotI sites. Phosphomimetic (Ser-666-Glu) and non-phosphorylatable (Ser-666-Ala) hTPC2 mutants were generated using the QuikChange site-directed mutagenesis kit (Stratagene, La Jolla, CA). Verified mutant constructs were inserted into a pΔMX-IRES-EGFP retroviral vector. Stable hTPC2-expressing cell lines and control EGFP-expressing line were generated by a retroviral expression system using a standard protocol. In brief, retroviral particles were produced by transfecting HEK293T cells with 5 μg of retroviral vector with the gene of interest, 4.5 μg of pUMVC (addgene #8449), and 0.5 μg of pVSV-G (addgene #8454) using the MegaTran transfection reagent (OriGene, Rockville, MD). Viral particles were collected at 48 and 72 hours after transfection. Inositol trisphosphate receptor deficient DT40TKO cells were infected by the retrovirus and the stably transduced cells were selected using flow cytometry by selecting for the GFP-positive cells. Stable cells were then expanded and frozen. Human TPC2 expression was assayed by western blotting and immunocytochemistry. In some experiments, C-terminal GFP-tagged hTPC2 was used. GFP-tagged hTPC2 was generated by sub-cloning hTPC2 into the pENTR1A-GFP-N2 vector (Addgene #19364) and the stable DT40TKO-hTPC2-GFP line was generated using a standard protocol.

### Western blot analysis

DT40TKO-hTPC2 and control DT40TKO-EGFP cells were washed with ice-cold phosphate-buffered saline (PBS). Cells were lysed in lysis buffer (50 mM Tris-HCl, 150 mM NaCl, 1% Triton X-100, pH 8.0) with protease inhibitors (Complete Protease Inhibitor Tablets, EDTA-free, Roche, Basel, Switzerland). Cell lysates were centrifuged at 13,200 x g at 4 °C for 30 min and the supernatant was collected. Proteins were separated using 10% SDS-PAGE gels and electrophoretically transferred to polyvinylidene difluoride membranes. Membranes were blocked by incubating the membrane for 1 hour in Tris-buffered saline containing 0.1% Tween-20 (TBST) with 5% non-fat milk at room temperature. Membranes were then incubated with anti-hTPC2 antibody (Cat#: Y158030; 1:500; Applied Biological Materials Inc., Richmond, BC, Canada) overnight at 4°C in TBST with 5% non-fat milk. Membrane was washed with TBST and incubated with HRP-conjugated anti-rabbit IgG secondary antibody (1:10,000, Bio-Rad Laboratories, Hercules, CA, USA) for 2 hours at room temperature in TBST with 5% non-fat milk. Chemiluminescence emitted upon the addition of HRP substrate (Millipore, Billerica, USA) was captured by X-ray film.

### Fluorescence microscopy

DT40TKO-hTPC2-GFP cells were placed on poly-D-lysine-coated coverslips and stained with 500 nM ER-Tracker Blue-White DPX (Invitrogen) for 30 min at 37 °C. The coverslips were then rinsed with PBS and replaced with fresh ER-tracker-free medium. Stained cells were visualized under a Nikon Eclipse Ti microscope using a 40x oil-immersion objective (Nikon CFI S Fluor Objective, Nikon, Tokyo, Japan). Fluorescence images of ER Tracker Blue staining and GFP fluorescence were captured using a SPOT RT3 CCD digital microscope camera (SPOT Imaging Solutions, Michigan, USA) and analysed using ImageJ (U.S. National Institutes of Health, Bethesda, Maryland).

### Measurement of NAADP-induced Ca^2+^ release in DT40TKO-hTPC2 and control DT40TKO-EGFP cells

NAADP was applied intracellularly in DT40TKO cells through a glass pipette in the whole-cell configuration, as previously described^9^. Briefly, cells seeded on poly-D-lysine-coated coverslips were incubated for 30 min at room temperature with 5 μM Fura-2-AM and 1% BSA in HEPES buffer (137 mM NaCl, 5.4 mM KCl, 0.25 mM Na_2_HPO_4_, 0.44 mM KH_2_PO_4,_ 2 mM CaCl_2_, 4.2 mM NaHCO_3_, 5.55 mM glucose, 10 mM HEPES, pH 7.4). After incubation, coverslips were washed in Fura-2-free HEPES buffer, placed in an imaging chamber (RC-21B, Warner Instruments, Connecticut, USA), and superfused with Ca^2+^ -free HEPES buffer for at least 5 min before experimentation. Patch pipettes with pipette solution (140 mM KCl, 10 mM HEPES, 1 mM MgCl_2_, 5 μM Fura-2, pH 7.4)^9^,^33^ containing 10 nM NAADP were used. The free Ca^2+^ concentration in pipette solution was 70 nM as determined by fluorometric method^34^. NAADP was then dialyzed to DT40TKO-hTPC2 and control DT40TKO-EGFP cells in the whole-cell configuration. Intracellular Ca^2+^ concentrations, reflected by the Fura-2 fluorescence ratio (*F*_340_/*F*_380_ excitation; emission 510 nm) were recorded at room temperature with a sampling frequency of 0.2 Hz using MetaFluor imaging software (Molecular Devices, CA, USA) and a SPOT RT3 CCD digital microscope camera (SPOT Imaging Solutions, Michigan, USA) at 40x magnification (Nikon, Tokyo, Japan). The seal resistance was monitored online using a HEKA EPC-10 amplifier (HEKA Elektronik Dr. Schulze GmbH, Germany). Experiments with seal resistance <1 GΩ were discarded.

### Isolation of nuclei and patch-clamp recording

Isolation of DT40TKO-hTPC2 nuclei for patch-clamp recordings were prepared by homogenizing cells in nuclei isolation solution (150 mM KCl, 250 mM sucrose, 10 mM Tris-HCl, 1.4 mM β-mercaptoethanol, 0.1 mM PMSF, Complete protease inhibitors, pH 7.3) as previously described^18^,^21^,^32^. In brief, cells were washed in ice-cold PBS and centrifuged at 300 × g for 5 min. The cell pellet was resuspended in an appropriate volume of ice-cold nuclei isolation solution. Resuspended cells were transferred to an ice-cold homogenizer (1-mL Duall homogenizer, Kimble-Chase, Vineland, NJ) and were subjected to 12 strokes of homogenization. Forty μl of cell homogenate were placed in 1 ml of bath solution (140 mM KCl or CsCl, 10 mM HEPES, 200 nM free Ca^2+^, 0.5 mM BAPTA, pH 7.3) and allowed to adhere to a glass-bottom culture dish for 10 min prior to electrophysiological experiments. Isolated nuclei were morphologically distinguishable from intact cells based on their unique morphology (Fig. 3a). The “on-nucleus” patch-clamp configuration was employed to detect the single hTPC2 channel using a HEKA EPC-10 amplifier (HEKA Elektronik) and pipets filled with pipette solution (140 mM KCl, 10 mM HEPES, 0.5 mM Na_2_ATP, 2 μM free Ca^2+^ buffered with 0.5 mM 5,5′-dibromo BAPTA, pH 7.3). Free [Ca^2+^] in solutions was adjusted by Ca^2+^ chelators with appropriate affinities and verified by fluorometry, as previously described^34^. Pipette resistances generally fell between 10 and 20 MΩ and seal resistances were >1 GΩ, respectively. NAADP was added directly to the patch pipette solution. In some experiments, 50 nM protein kinase A (PKA) catalytic subunit (Cat#: 539576, EMD Millipore Corp., Billerica, MA) was added to the pipette solution together with 10 nM NAADP. Single TPC2 channel traces were sampled at 5 kHz and filtered at 1 kHz. Only recordings that lasted for at least 30s were used for data analysis.

### Data analysis

Data were acquired using PATCHMASTER software (HEKA Elektronik). Single-channel current traces exhibiting one or two TPC2 channels were used for open probability (*P*_o_) analysis using QuB software^35^. All-points current amplitude histograms were generated from the current records and fitted with normal Gaussian probability distribution functions. The coefficient of determination (R^2^) for every fit was >0.95. Slope conductance was determined from linear fitting of current-voltage (*I/V*) relationships. Open and closed dwell times and burst analysis were performed using the Clampfit 10 Data Analysis Module (pClamp 10, Molecular devices, CA, USA). Channel dwell time constants for the open states were determined from mono-exponential fitting of the open dwell time histograms. Logarithmic plots of dwell closed times revealed two populations, with one larger than the other by an order of magnitude, which indicated the existence of multiple closed states. The burst delimiter (*T*_c_) was defined as 4 ms, as previously described^36^,^37^. Any closing time that was longer than *T*_c_ was defined as an inter-burst interval, whereas any time shorter than *T*_c_ was defined as closed time. These values were calculated after curve fitting of the histograms using the exponential logarithmic probability function. The coefficient of determination (R^2^) was determined for every theoretical curve fitting and considered acceptable when greater than 0.95.

## Additional Information

**How to cite this article**: Lee, C. S.-K. *et al.* Characterization of Two-Pore Channel 2 by Nuclear Membrane Electrophysiology. *Sci. Rep.* **6**, 20282; doi: 10.1038/srep20282 (2016).

## Supplementary Material

Supplementary Information
