# Supplementary material for: Characterization of Two-Pore Channel 2 by Nuclear Membrane Electrophysiology
Source: Sci Rep. 2016 Feb 3;6:20282. doi: 10.1038/srep20282 (PMC4738322; doi:10.1038/srep20282)
Supplement: Supplementary Information [file srep20282-s1.doc]

*Supplementary information for*

**Characterization of Two-Pore Channel 2 by Nuclear Membrane Electrophysiology**

Claire Shuk-Kwan Lee^1^, Benjamin Chun-Kit Tong^1^, Cecily Wing-Hei Cheng^1^, Harry Chun-Hin Hung^1^ and King-Ho Cheung^1,2, *^.

^1^School of Biomedical Sciences and ^2^Research Centre of Heart, Brain, Hormone and Healthy Aging, LKS Faculty of Medicine, University of Hong Kong, Hong Kong, China.

^*^To whom correspondence should be addressed:

School of Biomedical Sciences, LKS Faculty of Medicine, University of Hong Kong,

21 Sassoon Road, Pok Fu Lam, Hong Kong, China.

Tel.: +852-39179511; Fax: +852-28559730; email: [kingho.cheung@hku.hk](mailto:kingho.cheung@hku.hk)

*
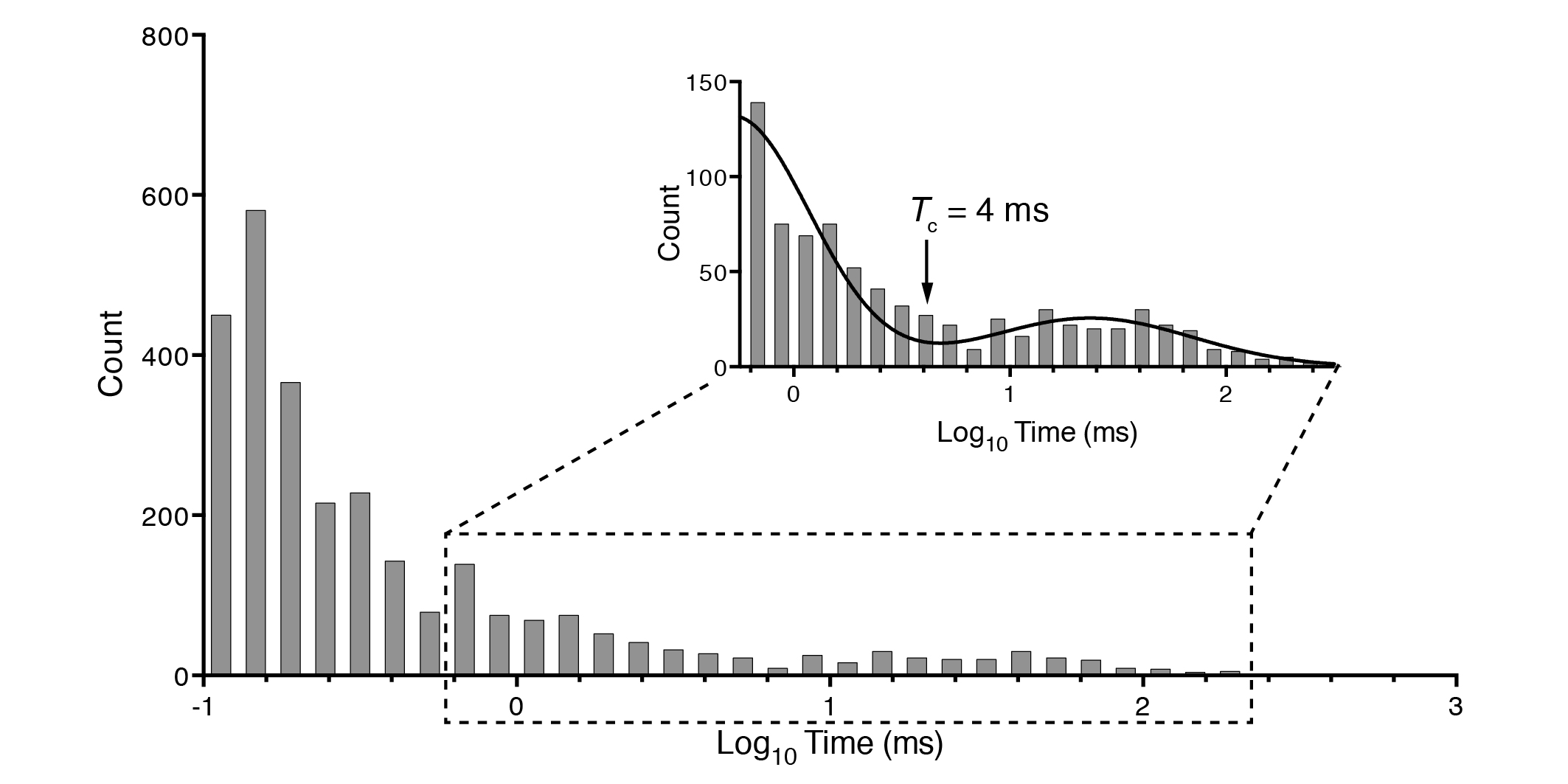
*

**Supplementary Fig. 1. Burst analysis of human TPC2 (hTPC2).** Closed-time histogram of hTPC2. Data were obtained from single-channel experiments in the presence of 100 nM NAADP. Data were plotted with a logarithmic x-axis and linear y-axis. Burst delimiter (*T*_c_) was defined as 4 ms, as described in the Methods section.


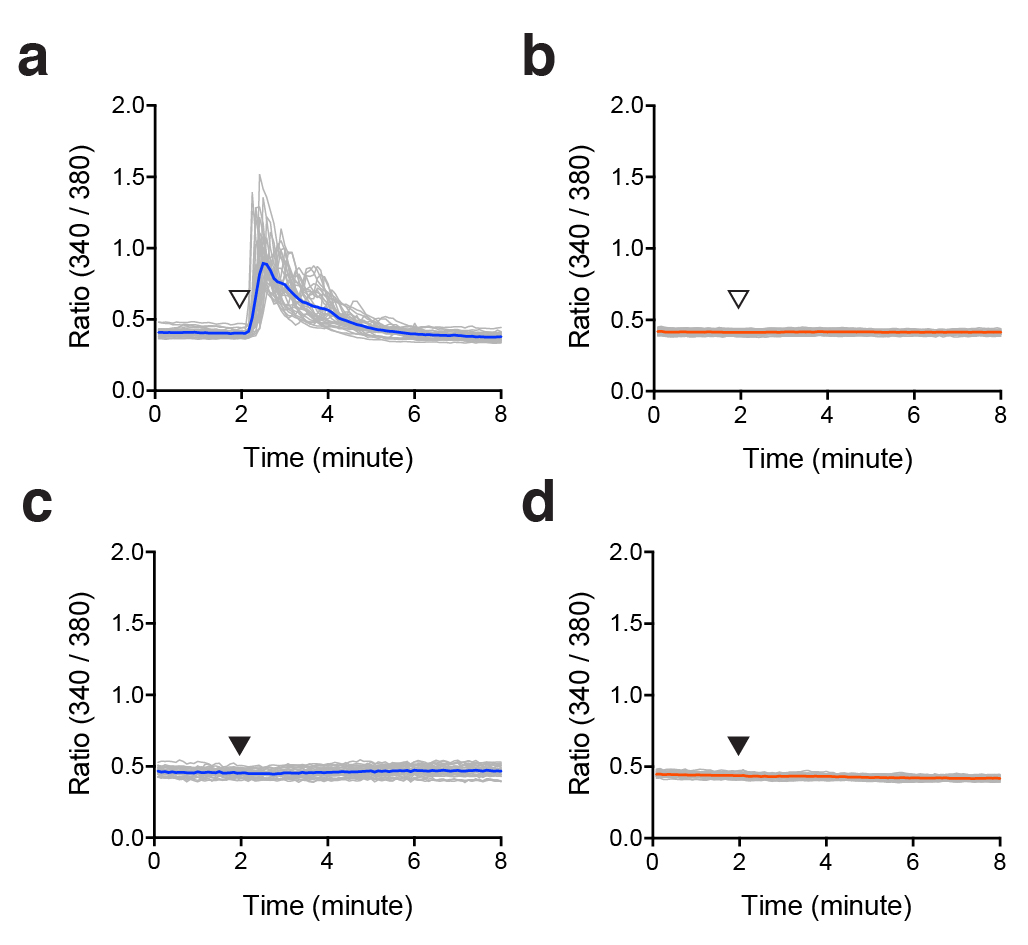


**Supplemental Fig. 2. Agonist-induced calcium response in wild-type DT40 and InsP_3_R deficient DT40 (DT40TKO) cells.** Representative imaging results in wild-type DT40 cells depicted calcium response stimulated by (a) anti-IgM antibody (50 µg/ml) or (b) caffeine (2 mM). Representative imaging results in DT40TKO cells depicted calcium response stimulated by (c) anti-IgM antibody (50 µg/ml) or (d) caffeine (2 mM). Grey lines showed the calcium response of individual cells while the blue and red lines summarized the average calcium response in respond to the stimulation of anti-IgM and caffeine, respectively. Each agonist stimulation experiments were repeated 3 times and 30 cells were analysed in every individual experiment. Arrow heads indicated the time for agonist addition.

**
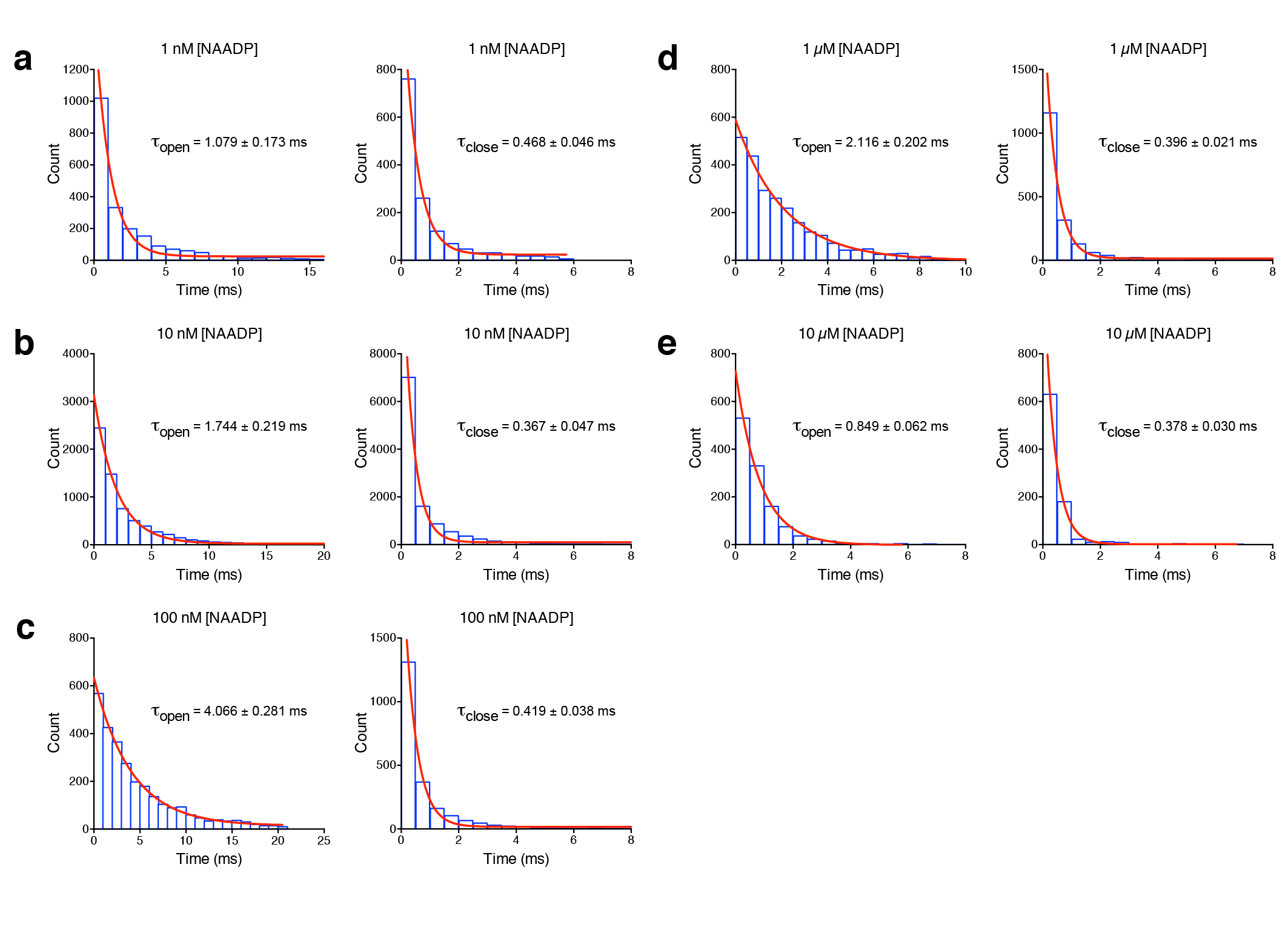
**

**Supplemental Fig. 3. NAADP regulated hTPC2 channel *P*_o_ by modulating the channel open time.** Histograms summarised the dwell time analyses of mean open (left) and closed (right) time at different NAADP concentrations (a to e). Trace was summarized from 3 individual patches in each NAADP concentration. Time constants are inserted into each graph.


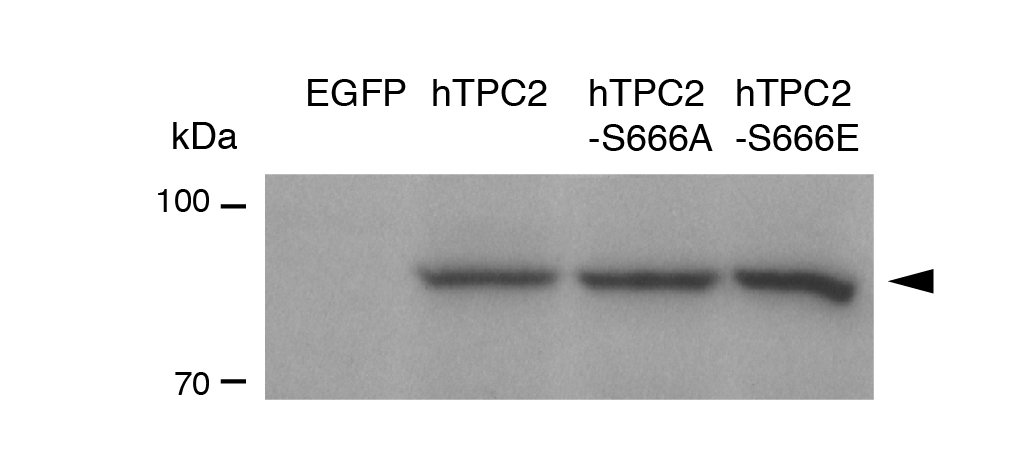


**Supplementary Fig. 4. Expression of phosphomimetic and unphosphorylatable hTPC2 mutants in DT40TKO cells.** Western blot revealed hTPC2 expression in DT40TKO-EGFP (lane 1), DT40TKO-hTPC2 (lane 2), DT40TKO-hTPC2-S666A (Lane 3), and DT40TKO-hTPC2-S666E (Lane 4) cells. Twenty-five µg of cell lysate were loaded in each lane and arrowhead indicates the immunoreactive band of hTPC2.

**
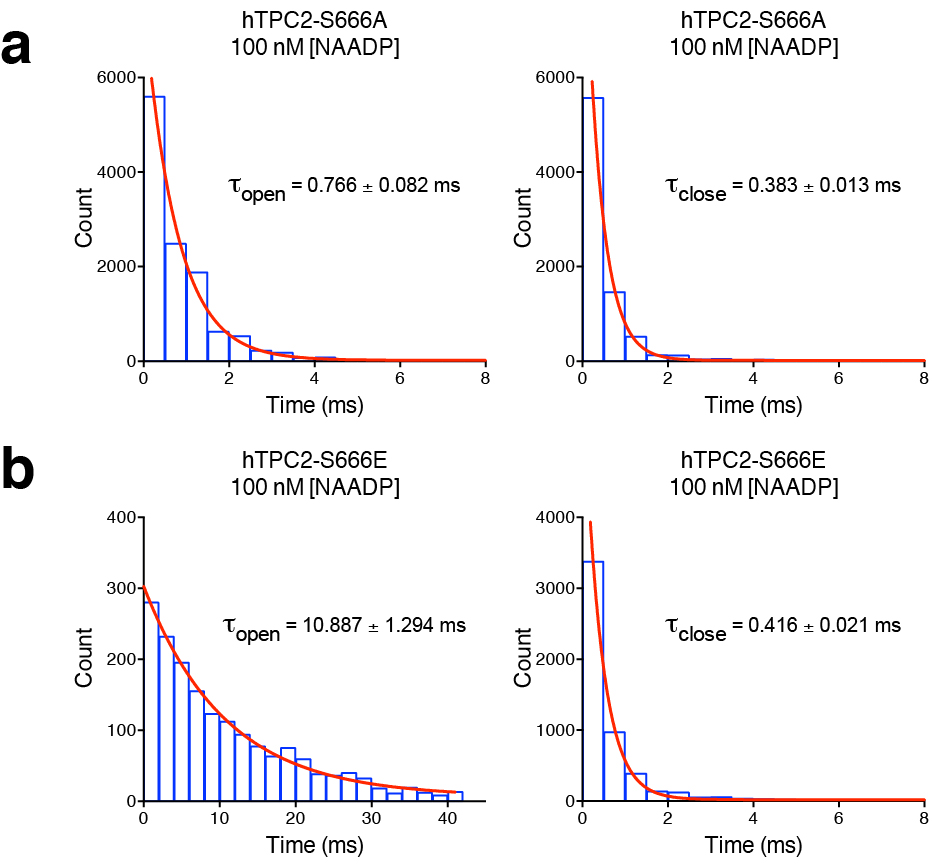
**

**Supplemental Fig. 5. PKA phosphorylation regulated hTPC2 channel activity by modulating the channel open time.** Histograms summarised the dwell time analyses of mean open (left) and closed (right) time histograms of unphosphorylatable (a) and phosphomimetic (b) mutants. Trace was summarised from 3 individual patches in each mutant line. Time constants are inserted into each graph.
